# High-prevalence and broad spectrum of Cell Adhesion and Extracellular Matrix gene pathway mutations in epithelial ovarian cancer

**DOI:** 10.1186/2043-9113-2-15

**Published:** 2012-09-24

**Authors:** Arash Rafii, Najeeb M Halabi, Joel A Malek

**Affiliations:** 1 Stem cell and microenvironment laboratory, Weill Cornell Medical College in Qatar, Education city, Qatar Foundation, Doha, Qatar; 2 Department of Genetic Medicine, Weill Cornell Medical College, NY, NY, USA; 3 Genomics Core, Weill Cornell Medical College in Qatar, Education city, Qatar Foundation, Doha, Qatar; 4 Department of Genetic Medicine, Weill Cornell Medical College, Genomics Core Laboratory, Weill Cornell Medical College in Qatar, Qatar-Foundation, Doha, Qatar

## Abstract

**Background:**

Ovarian cancer is the most deadly gynecological cancer because of late diagnosis, frequently with diffuse peritoneal metastases. Recent findings have shown that serous epithelial ovarian cancer has a narrow mutational spectrum with TP53 being the most frequently targeted when single genes are considered. It is, however, important to understand which pathways as a whole may be targeted for mutation.

**Findings:**

Previously published mutational data provided by the cancer genome atlas networks findings on ovarian cancer was searched for statistically significant enrichment of genes in pathways. These pathways were then searched in all patients to identify the spectrum of mutations. Statistical significance was further shown through in-silico permutations of exome sequences using empirically observed mutation rates. We detected mutations in the cell adhesion pathway genes in more than 89% of serous epithelial ovarian cancer patients. This level of near universal mutational targeting of the cell adhesion pathway, including the extracellular matrix pathway, is previously unreported in epithelial ovarian cancer.

**Conclusions:**

Taken together with previous studies on the role of cell adhesion and extracellular matrix gene expression in ovarian cancer and metastasis, our results identify pathways for which the mutational prevalence has previously been overlooked using single gene approaches. Analysis of mutations at the pathway level will be critical in studying heterogeneous diseases such as ovarian cancer.

## Background

Epithelial Ovarian Carcinoma (EOC) is the sixth most common malignancy in women [1] and has a poor overall survival rate (20 to 30% at 5 years). High-grade serous carcinoma is the most frequent type of ovarian cancer. The poor survival rate is due mainly to a large tumor burden and frequent, extensive peritoneal metastatic lesions at diagnosis. This results in difficulty to achieve complete optimal resection, despite advances in surgical practice. Indeed the importance of the metastatic process in ovarian cancer has been clearly demonstrated in the clinical setting by the fact that peritoneal residual disease at the end of the surgical treatment (even below 5 mm) impacts prognosis [2,3].

Despite initial chemosensitivity and ultra-radical debulking surgery most patients will present with diffuse peritoneal recurrences rather than distant metastasis. Therefore, understanding the molecular mechanisms of progression from primary to metastases is critical for the development of effective therapies. Most studies of EOC have focused on the primary tumor including mutational and gene expression analysis. Many transcriptomic studies have been performed demonstrating different gene expression signatures depending on the histologic subtypes, the grade and stage [4-6]. Others have defined several prognosis signatures however there is only little overlap between these studies [7-9].

Recent results from the TCGA group’s exome sequencing of 316 epithelial ovarian cancer primary tumors with matched controls revealed a very narrow spectrum for somatic mutations in EOC. Specifically, TP53 was mutated in approximately 96% of EOC primary tumors. The next most frequently affected genes were mutated in less than 6% of tumors. This dramatic prevalence of TP53 mutations suggests an early and central role of TP53 mutations in EOC.

In our recent studies of copy number variations and gene expression differences between primary and metastatic lesions [10,11] we observed clear targeting of pathways, rather than specific genes in EOC. Indeed our findings showed that analysis of pathways reduced the overall heterogeneity in comparisons and this pathway-based approach may also be important in studying the mutational spectrum of ovarian cancer as well. The importance of pathway-based analysis over single gene analysis is due to the fact that similar downstream effects can be obtained by mutation of different genes within the same pathway. The TCGA study included some discovery-based pathway analysis on copy number and gene expression data using the PARADIGM approach. Analysis of mutations, however, was mainly restricted to certain known cancer pathways. The HOTNET approach overlaid the mutational data on protein interaction networks but no further discovery-based pathway analyses of gene mutations were reported [12].

We therefore hypothesized that analyzing the recent mutational data in EOC [12] using a broader functional pathway approach such as the Gene Ontologies or KEGG could reveal consistent targeting of pathways other than known cancer pathways, and could reduce the observed heterogeneity when only individual genes are considered. Furthermore, analysis of low frequency mutated genes within frequently mutated pathways, may offer insight into the metastatic process that only a few clones within the primary tumor undertake, but for which many patients are susceptible to.

## Methods

Data was obtained from the TCGA study’s supplementary information where a total of 316 EOC tumors were subjected to exome sequencing and the predicted mutations reported [12]. We selected genes that contained predicted non-silent mutations in at least 3 patients (mutated in at least ~1% of patients). These genes were searched for enrichment in KEGG pathways and Gene Ontologies using DAVID [13] and GeneTrail [14]. These software packages offer robust statistical testing with appropriate multiple-hypothesis testing correction.

*In-silico* random mutagenesis was conducted using a sequence library comprising exons that overlap with the probes used in Agilent’s All Exon 50 MB kit. Custom scripts were written to introduce random mutations into these sequences and check for *in-silico* mutation rates in specific pathways. We used the mutation rates observed by the TCGA study: an AT mutation rate of 8.54 x 10^-7^, 1.2x10^-6^ for all GC bases, and 4.31 x 10^-6^ for CpG repeats. Since insertion/deletions are not sequence specific, we added the rate of insertions/deletions 2.2x10^-7^ to the other mutation categories. 100 permutations were conducted where each permutation consists of 316 random mutation runs mimicking the number of tumors analyzed by the TCGA. Data was gathered from the permutations to offer an empirically observed statistic.

To determine a pathways mutational spectrum and prevalence, each tumor’s data was queried for potentially deleterious mutations in any genes containing a Gene Ontology annotation of ‘Cell Adhesion’ in the ‘Molecular Function’ category. All occurrences were recorded and summed across the pathway, for mutational spectrum, and set of patients for pathway mutation prevalence.

## Results

Genes predicted to be mutated in at least 3 patients resulted in a list of 1382 genes that were most significantly enriched for Gene Ontology category “Cell Adhesion” (Benjamini-Hochberg adjusted p-value = 1.03x10^-25^) with 156 genes identified. This was ~2.5 times more than 60 genes expected by random permutation. In KEGG, top enriched pathways included ‘Extracellular Matrix (ECM)-receptor interaction’ (BH p-value = 3.35x10^-11^) and ‘Focal Adhesion’ (BH p-value = 2.62x10^-9^) with 30 genes (4.5x the expected 6.5 genes) and 45 genes (2.9x the expected 15.5 genes) respectively. Both extracellular matrix and focal adhesion pathways share many of the same genes. Other pathways including ‘Calcium signaling’ were significantly enriched though less so than those mentioned above.

To more accurately estimate the significance of the TCGA observed mutations within the cell adhesion genes, we carried out an *in-silico* random mutagenesis simulation. If the frequency of random simulated mutations in cell adhesion genes is significantly less than the frequency of observed mutations in cell adhesion genes, this could suggest that there is selection in the tumor for cell adhesion mutations.

**Figure 1:**
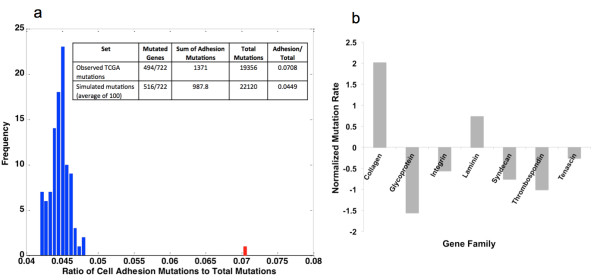
**Simulated and observed Extracellular matrix gene family mutations in ovarian cancer.** (**a**) Histogram showing the ratio of mutations in cell adhesion genes in the observed dataset (in red) and the ratio of mutations in cell adhesion genes to total mutations in simulated mutagenesis data (in blue). (**b**) Mutation rates were normalized to gene numbers in the family and compared to the baseline of all mutations in all genes. Collagens and Laminins had higher than expected mutation rates while the large gene family of Integrins had lower mutation rates.

We ran 100 replicates of a trial where each trial consists of 316 random mutation runs mimicking the number of tumors analyzed by the TCGA. It is important to point out that this approach assumes equal mutational opportunity for the entire 50 Mb capture region while typically only a portion of the targeted region is captured. This may result in some bias of the model. Nevertheless, our results (see Figure 1a), show that the frequency of simulated cell adhesion gene mutations in all 100 trials is significantly less (empirical p-value less than 0.01) than the frequency of observed cell adhesion gene mutations. This suggests that there is indeed a higher mutation rate in cell adhesion genes in actual ovarian tumors.

Upon identifying cell adhesion genes as being highly enriched for mutations in the TCGA data, we determined the spectrum and the prevalence of mutations in this functional category among all patients. Each tumor’s data was queried for all mutations, other than those predicted to be silent, in any of the 576 genes containing a Gene Ontology annotation of ‘Cell Adhesion’ in the ‘Molecular Function’ category. We found a broad spectrum of mutation in this category with 366 (64%) of genes having a potentially deleterious mutation in at least one tumor (Table 1).

**Table 1:** Pathways enriched for non-synonymous SNPs in the TCGA data

	**Molecular Function/Pathway**	Enrichment Rank by p-value*	**Enrichment P-value (BH corrected)**	**Enrichment level (observed/expected)**	**Pathway mutational spectrum:% of genes**	**Pathway Mutational Prevalence: % of tumors**	**# genes mutated in at least 3 patients**
KEGG	ECM-receptor Interaction	1	3.35x10^-11^	~4.5X	74% (62/84)	40% (127/316)	30
	Focal Adhesion	2	2.62x10^-9^	~2.9X	(61%) 122/199	58% (183/316)	45
	Calcium Signaling	3	2.05x10^-8^	~2.9X	63% (112/179)	48% (153/316)	40
Gene Ontology	Cell Adhesion	1	1.03x10^-25^	~2.5X	64% (366/576)	89% (281/316)	156
	Developmental process	14	1.03x10^-15^	~1.5X	49% (430/869)	90% (284/316)	375
	Extracellular Matrix	19	2.56x10^-14^	~2.8X	53% (80/150)	40% (127/316)	76

*Enriched pathways from all genes with predicted mutations in at least 3 ovarian tumors from the TCGA data were ranked by pvalue and selected pathways reported here.

We further calculated the prevalence of ‘Cell Adhesion’ mutations among all patients’ tumors. Strikingly, 281 of the 316 (89%) tumors investigated had a mutation in at least one cell adhesion gene while 207 (66%) tumors had mutations in at least two cell adhesion genes. This number of tumors with a mutation in the cell adhesion molecular function is comparable to the 283 automatically identified mutations in TP53. Results from other pathways are summarized in Table 1.

We also observed selection of certain gene families within functional categories that were targeted for mutation while other gene families were avoided. We identified all mutations in the KEGG pathway ‘Extracellular Matrix (ECM)-receptor interaction’ and normalized the number of mutations to the number of genes in selected gene families. A baseline ratio of all mutations to genes within the KEGG pathway was subtracted from each gene family (Figure 1b). We observed that collagens and laminins are especially targeted for mutation having ~2 and ~1 more mutations per gene than the baseline. Other categories including the integrins acquired fewer mutations than the average (Figure 1b).

## Conclusions

The mutational spectrum in epithelial ovarian cancer is very narrow when only single genes are considered. TP53 mutations are near universal [12] with few other genes rising to significance. However, little work has been done to identify potential mutational targeting of pathways other than standard cancer pathways. Taken together, our data suggests that the spectrum of mutations in cell adhesion related genes is broad across this molecular function, with 64% of cell adhesion genes mutated at least once, and most interestingly with 89% of tumors observed to have at least one mutation.

The observations presented here are consistent with several reported studies on cell adhesion and tumorigenesis. Cell adhesion and extracellular matrix are known to be critical pathways in the metastatic process [15-17] and our results suggest there may be multiple genes, within the same pathway that EOC uses to achieve tumorogenic capability. Targeting of cell adhesion related genes agrees well with multiple previous reports of EOC specific gene expression changes in this pathway [18,19]. However, the breadth and prevalence of the mutational targeting of this pathway is a novel finding. Moreover, our results confirm recent findings that the ECM receptor interaction pathway is affected in ovarian cancer specifically and in many other cancers in general. In a recent study of cancer genetic deregulation at the transcriptomic level, the cell adhesion pathway was the most conserved for deregulation, showing enrichment in 26 tumor types [20]. In the same study, the ECM-receptor interaction pathway was found highly deregulated in many cancers including ovarian cancer.

The deregulation in the cell-adhesion and ECM pathway has broad implications. Indeed a study of EOC gene expression revealed agreement on an increase in ECM gene expression in chemotherapy resistant tumors [21]. Moreover, molecular subtypes of tumors, based on gene expression studies, have been identified and the categories encompassing high-grade serous EOC regularly include upregulated expression of ECM and cell adhesion genes [22]. A recent study of CNV in EOC primary tumors revealed an increase of extracellular matrix and cell adhesion genes in tumors with no CCNE1 amplification, yet with a short time to relapse [23]. Gene expression analysis of cisplatin resistant cancer cells revealed extracellular matrix related genes as a primary differentiator of chemotherapy resistance and Collagen 6A3 (COL6A3) has been shown to contribute to cisplatin resistance [24]. Our analysis agrees well with previous findings by Capo-chichi and colleagues that collagen IV and laminin are aberrantly expressed in primary ovarian tumors [25].

The importance of extracellular matrix and cell adhesion genes in EOC and other cancers is clear [26,27]. Other groups’ gene expression studies, and our own analysis of both the prevalence and broad spectrum of mutation within cell adhesion gene groups reinforce the likely central role cell adhesion plays in epithelial ovarian cancers.

## Competing interests

The authors declare that they have no competing interests.

## Authors' contributions

AR envisioned, analyzed data and helped write the manuscript. NMH analyzed data and helped write the paper. JAM envisioned the study, analyzed data and wrote the manuscript. All authors read and approved the final manuscript.
